# Elder Abuse: A Comprehensive Overview and Physician-Associated Challenges

**DOI:** 10.7759/cureus.14375

**Published:** 2021-04-08

**Authors:** Karan Patel, Sean Bunachita, Hannah Chiu, Prakul Suresh, Urvish K Patel

**Affiliations:** 1 Medicine, Cooper Medical School, Camden, USA; 2 Molecular and Cellular Biology, Johns Hopkins University, Baltimore, USA; 3 Radiation Oncology, The University of Texas MD Anderson Cancer Center, Houston, USA; 4 Public Health and Neurology, Icahn School of Medicine at Mount Sinai, New York, USA

**Keywords:** elder abuse

## Abstract

Elder abuse can present in many forms, including physical abuse, psychological/emotional abuse, sexual abuse, financial abuse, and neglect. Many studies estimate that about 10% of all people over the age of 65 experience some form of abuse. These rates are often higher in long-term care facilities such as nursing homes, despite government regulations aimed toward addressing this issue. Because patients who experience abuse tend to have higher rates of hospitalization and mortality, it is important for physicians to be able to accurately identify cases of abuse. However, many studies have found that healthcare professionals are often undertrained and ill-equipped in diagnosing elder abuse. In this article, we outline tools that may be able to aid healthcare professionals in their diagnoses, such as survey-based methodology and common physical signs of abuse. In addition, we propose evidence-based solutions, including the use of multidisciplinary teams and increased training on the subject, so that healthcare professionals can more easily identify victims of abuse. Essentially, it is our hope that this article further spotlights elder abuse and its challenges, while serving as a guide to healthcare professionals.

## Introduction and background

The World Health Organization (WHO) defines elder abuse as, “a single, or repeated act, or lack of appropriate action, occurring within any relationship where there is an expectation of trust which causes harm or distress to an older person" [1]. Unfortunately, elder abuse is prevalent around the world, with studies finding that 10% of all people over the age of 65 experience some form of abuse, with rates still continuing to rise [2,3]. Despite these alarming numbers and trends, it is estimated that only 1 in 14 cases of elder abuse are reported to the correct authorities [4]. Elder abuse can result in serious psychological and physical consequences for victims. Studies have shown that victims of abuse are almost three times as likely to suffer hospitalizations than their counterparts; victims of abuse have also been shown to have significantly higher mortality rates [5,6]. Physicians and other health care providers are among the few that may have an opportunity to intervene when someone is being abused. While taking a history and conducting a physical exam, physicians may be able to detect signs of subtle underlying abuse that a victim may cover up in a normal setting. However, physicians are often under trained and unconfident in their ability to identify abuse in [7]. As a result, many preventable cases of elder abuse go unnoticed each year.

## Review

Forms of elder abuse

Elder abuse comes in various forms which include: (1) physical abuse, (2) psychological/emotional abuse, (3) sexual abuse, (4) financial abuse, and (5) neglect. Physical abuse is defined as any intentional act that results in harm to a person. Psychological or emotional abuse includes verbal threats, harassment, intimidation, and isolation. Sexual abuse occurs when a victim either is forced into a non-consensual act or is incapable of consenting to such an action. Examples of this include, but are not limited to, rape, forced nudity, and inappropriate touching [8]. Financial exploitation occurs when the abuser is controlling and misusing the victim’s financial accounts. The misuse can include actions such as changing a will, stealing from bank accounts, and performing financial transactions that are not in the best interest of the victim [9,10]. Finally, neglect pertains to situations in which a caretaker does not adequately fulfill their duties; this includes such actions as not taking an elder to their doctor’s appointments and not assisting them with daily activities they cannot do themselves, such as personal hygiene maintenance [8]. A study conducted by Acierno et al. found that the most common types of abuse experienced by their sample were financial abuse, neglect, emotional abuse, physical abuse, and sexual abuse, in that order [11]. Nonetheless, the prevalence of each type of abuse has yet to be fully determined, though some studies argue that neglect is actually the most common form of abuse [10,12]. The lack of a clear answer may be a result of variations in settings and methods between studies. As stated previously, elder abuse, in general, is associated with an increased risk of hospitalization and death. One study found an elevated risk of death in patients who experienced elder mistreatment compared to those who did not (odds ratio: 3.1, 95% confidence interval: 1.4-6.7) [6]. Another study by Dong et al. found an increased risk of hospitalization (odds ratio: 1.97, 95% confidence interval: 1.33-2.61) among victims of abuse [5]. One thing to note is that rates of hospitalization vary among the different subcategories of abuse. Psychological abuse and neglect were most associated with increased hospitalization, while financial abuse was ranked lower among abuse-related risk factors [5].

Abuse in long-term care facilities

Nursing homes and other long-term care facilities are among the places with the highest rates of elder abuse. Widespread concern over this first became apparent in the 1970s when there were almost no federal regulations for these facilities [13]. In 1986, the Institute of Medicine, at the request of Congress, conducted a study in which they found high rates of abuse and neglect among nursing home residents. In 1987, the Nursing Home Reform Act (NHRA), as part of the Omnibus Budget Reconciliation Act, was passed in order to help ensure the overall well-being of residents through federal regulations. The act included laws such as providing Medicare and Medicaid payments to nursing homes only if they complied with government requirements [14]. The main commandments set forth by the NHRA were tenfold: residents of nursing homes had “(1) the right to freedom from abuse, mistreatment, and neglect, (2) the right to freedom from physical restraints, (3) the right to privacy, (4) the right to accommodation of medical, physical, psychological, and social needs, (5) the right to participate in resident and family groups, (6) the right to be treated with dignity, (7) the right to exercise self-determination, (8) the right to communicate freely, (9) the right to participate in the review of one's care plan and to be fully informed in advance about any changes in care, treatment, or change of status in the facility, and (10) the right to voice grievances without discrimination or reprisal” [14].

Despite government efforts, elder abuse in nursing homes continues to remain a major problem [15]. The main types of abuse in nursing homes are as follows: physical abuse (29%), resident-to-resident abuse (22%), gross neglect (14%), financial abuse (7%), and sexual abuse (7%) [16]. A study conducted by Hawes et al. found that 40% of the staff in their sample reported committing at least one instance of psychological abuse over a 12-month period. These actions included, but were not limited to, yelling and swearing at residents, inappropriate isolation, and denying food privileges [17]. Another study found that 50% of the nursing home staff admitted to mistreating older patients, 17% of certified nursing assistants (CNAs) reported pushing, shoving, or grabbing a nursing home resident, 23% of CNAs reported swearing at residents, and 51% reported yelling at residents [16]. These numbers were significantly higher when residents were interviewed about their own experiences. In a study of over 2000 nursing home residents, 44% said they had been abused and 95% said that they had either themselves been neglected or seen another resident be neglected [16]. While the actions of abusers should never be justified, and we by no means condone them, they may be partially explained by the fact that nursing home staff and healthcare professionals are overworked and understaffed, which may contribute to their growing frustrations. They may then take these frustrations out on the residents in a stint of misplaced anger [18]. One study found that up to 90% of nursing homes are understaffed, and one nurse’s aide may have to take care of up to thirty patients at once. This is in spite of recommended guidelines for the nursing aide to resident ratio, which typically ranges from 1:3 to 1:6 [15]. As a result, nursing home reforms, including hiring more employees, may be a critical step to reduce rampant abuse.

Risk factors for elder abuse

In Table 1, we list factors that are commonly associated with perpetrators and victims of abuse. While this is by no means an exhaustive list, it may serve as a guide to aid in the diagnosis of elder abuse. One thing of note is that there are studies that have found conflicting results with some common risk factors. For example, while many studies cite co-inhabitance as a risk factor for abuse, a study conducted by Li et al. found that living alone was actually a risk factor for abuse [19]. Meanwhile, another study by Pérez-Cárceles et al. found that co-inhabitance was a risk factor only if the perpetrator had a mental health disorder [20]. We cite these studies here to remind readers that risk factors should only serve as a starting point and cannot solely be used to raise clinical suspicion for abuse. 

**Table 1:** Victim and perpetrator risk factors.

Victim risk factors	Perpetrator risk factors
Female [21]	High levels of stress [22]
Dementia [23]	History of alcohol abuse [22,24]
Abuser dependency [21]	History of drug abuse [22]
Social isolation [21]	Diagnosis of mental illness [24]
Physical disabilities [23]	Lack of social support [22]

Diagnosing elder abuse

It is important for elder abuse to first be accurately detected and diagnosed so that swift action can then be taken to intervene before the patient faces any further mistreatment. Yet, studies have shown that healthcare professionals are often inadequately equipped to identify cases of abuse. A US national survey of Emergency Department physicians found that 74% were uncertain whether elder abuse had been clearly defined and characterized, while 58% of physicians lacked confidence in their ability to correctly identify abuse in elderly patients [25]. This is an especially vital consideration since symptoms of elder abuse often mimic those found in other medical conditions. For example, burns can be mimicked by contact dermatitis while a bone fracture can be a symptom of osteoporosis [26]. Thus, it is crucial that healthcare providers are able to accurately differentiate between abuse and other conditions so that the appropriate treatment plan can be established. Below are a number of resources and methods that have been typically used to aid with the diagnosis of elder abuse. 

Patient history

An essential first step to identifying elder abuse is to ascertain the patient’s history in order to uncover potential risk factors and signs of current abuse. During this time, the healthcare provider should take note of patterns indicating abuse, such as lack of attendance for follow-up appointments, high frequency of injuries, and failing to promptly treat illness or injury [9]. It is crucial to interview the patient in a private setting, without any relatives or caregivers present, as the abuser(s) may be among that group. Additionally, it is important that the patient is as honest as possible in their answers, and they may be hesitant to share details of abuse if others are in the room [13]. Physician trust can be further gained by being sympathetic and nonjudgmental of the patient’s answers [13]. A number of screening tools have also been developed to supplement the history-taking process. These are generally a series of standardized questions either asked to or completed by the patient and help guide the physician to the next course of action. Three examples of common screening tools are shown next. 

Screening tools

Elder abuse suspicion index: The Elder Abuse Suspicion Index (EASI) is a two-minute screening tool that features a questionnaire of six dichotomous yes/no survey items, with five answered by the patient and one answered by the physician. When administered to cognitively intact patients 65 years of age or older, the assessment was found to have a sensitivity of 47% and a specificity of 75% if at least one EASI item was answered “yes” [27]. The EASI instrument possesses a number of advantages: (1) it can be conducted in a short time span, (2) it has been validated for both the English and French languages, and (3) it has been validated in a primary care setting [27,28]. However, it also contains some limitations: it can only be used for cognitively intact elderly patients seen by primary care physicians and it does not offer an evaluation of patient caregivers [29]. 

Elder assessment instrument: The Elder Assessment Instrument (EAI) is a 44-item screening tool that includes evaluations of social habits, medical history, and emotional/psychological neglect in the patient, among other questions. Each question is scored on a Likert scale ranging from 1 (no evidence) to 4 (evidence) [30]. The questionnaire takes approximately 15 minutes to complete and has a sensitivity of 71% and a specificity of 93% when administered by emergency department nurses [28,29]. A few advantages of the EAI are that it is relatively simple to administer, it has been validated in both the English and Spanish languages, and it has been validated for administration by nurses [30]. Some drawbacks of the assessment are its lack of an overall scoring system and lack of validation in subcategories [31]. 

Older adult psychological abuse measure: A third screening tool is the Older Adult Psychological Abuse Measure (OAPAM). The OAPAM is self-administered and specifically assesses psychological abuse by examining the level of risk in factors including isolation, insensitivity and disrespect, shaming and blaming, and reported threats and intimidation, as well as a category for other trusted risk factors [29]. In subjects who had already experienced an instance of violence and were found to be cognitively intact (as assessed by the Mini-Mental State Examination, or MMSE), one study found that the OAPAM showed 92% reliability [29,31]. However, the study was limited in that it only included participants located in Chicago, Illinois, so additional validation studies will need to be conducted in other populations. 

Physical exam

Following suspected elder abuse, it is imperative to conduct a comprehensive physical examination by a trained healthcare professional to uncover further evidence. The exam should encompass the patient’s entire body with a specific focus on detecting signs of abuse. Some common physical signs of mistreatment include welts, bite marks, fractures, dehydration, sexually transmitted infections, and poor hygiene [32]. It is also important to observe interactions between the patient and caregiver, looking for signs of anxiety or poor eye contact in the patient [9]. If possible, any findings should be further investigated and confirmed through laboratory testing. Some indications of abuse that can be detected through lab screening are dehydration, malnutrition, low medication levels, and drug poisoning [9]. 

Elder abuse reporting and treatment

Nearly all states, with the exception of New York, have laws stipulating mandatory reporting by healthcare professionals even under merely the suspicion of elder abuse [13]. This typically entails notifying a government regulatory agency, such as the Adult Protective Services (APS), who would then assign a social worker to investigate claims of elder mistreatment. If the patient is deemed to be in a sufficient cognitive state to make their own decisions, then they are free to decline further aid if they desire [32]. Should social assistance continue, then subsequent interventions tend to involve the cooperation of many different support systems throughout the community over long time periods [13]. While physicians often play a critical role in initially detecting elder abuse, they lack the amount of free time required to fully sustain follow-up and treatment alone. Thus, successful care must involve the interplay and coordination of a multidisciplinary team of trained professionals [13]. 

The ultimate goal of treating elder abuse is to ensure that every facet of the patient’s well-being is promptly addressed and that further mistreatment is prevented. Physical injuries are the first priority to reduce bodily pain and increase the quality of life [9]. Thereafter, the team should manage other forms of abuse, such as psychological, social, or financial. This could involve a number of interventions, such as mental health services, home health care, and meal delivery [13]. In the end, it is up to the discretion of the interprofessional team to determine the optimal treatment plan for each patient’s unique situation. 

Despite the legal requirements and resources available for reporting elder abuse, it has still been found to be the least reported type of domestic violence [33]. Physicians and other healthcare professionals are hesitant to report elder abuse for a myriad of reasons. They may believe that patients would be moving from one unwanted environment to another: that of understaffed and unsatisfactory care facilities [34]. Another concern for physicians is being sued for malpractice, especially if the physician reports suspected abuse when it is not actually occurring [35]. Further, elder abuse may not be reported due to a lack of training on how to do so. For example, a survey of Emergency Medical Services (EMS) providers discovered that there was an absence of protocols specific to reporting concerns in vulnerable elderly populations [36]. Finally, if the abuse is perpetrated by a close relative, health professionals may view the issue as a family matter that they should not intrude upon [33].

Potential challenges and associated solutions

Elder abuse persists due to the lack of awareness and education made available to key professionals. In order to bridge the gaps in detection and reporting, it is necessary to increase the hours of training on this topic for medical professionals. The majority of physicians reported their training on elder abuse to be not very adequate or not adequate at all, with most reporting no more than 10 hours of training. Additionally, two-thirds of residency programs fail to prioritize formal elder abuse training, and half of the physicians surveyed report having no residency training in elder abuse detection at all [4,37]. Thus, the best strategy to combat the lack of awareness is to emphasize and increase the training that is done in medical residency programs, making the requirement for elder abuse training a part of the curriculum [38]. It has been demonstrated that physicians who were offered continuing medical education (CME) for elder abuse were less likely to ignore the abuse of their patients as a barrier to reporting [4]. 

Furthermore, as mentioned previously, nursing homes show high rates of abuse, with one study of elders in nursing facilities finding that 44% of respondents had been subjected to prior mistreatment [39]. As with physician training, a key step to preventing elder abuse is to increase the education of nursing home caretakers and staff-members. These onsite training sessions teach caretakers how to utilize assistive equipment and raise awareness through discussing what to expect as individuals age, their mental health, and the specific illnesses of individual residents. A handful of nursing homes also prioritize educating residents and their family members, planning lighthearted activities that remind elders of the rightful treatment they should expect to receive [40]. Beyond this, many nursing homes purposefully remind residents of their safety in an overt way. They help facilitate a relationship between elders and the local police department and make crime prevention and abuse hotlines readily available for staff and residents. Many also have security systems in place and take the initiative to secure the residents’ valuables [40]. Collectively, these practices contribute to creating a safer environment. 

Additionally, many elders experience little relief or justice after facing financial abuse. This is partly due to the reluctance or inability of the elder to testify - but even if a victim is willing to prosecute, they can experience significant obstacles along the way. These include a high standard of proof for evidence that abuse has occurred, a lack of professional knowledge, and communication difficulties if the patient is cognitive- or speech-impaired [41]. One strategy to streamline the process of prosecution is to utilize multi-disciplinary teams, which include professionals within the justice system, physicians, nurses, mental health workers, and protective services. Having this array of professionals allows access to resources like neuropsychological testing, medical records, and legal services, which increase the likelihood of the case being reviewed, charged, and successfully prosecuted [42].

While these solutions have been proven to be effective, there are some limitations to them. Increased training during residency can require multi-disciplinary collaboration and agreement within each individual institution, and redeveloping parts of the curriculum will require thorough planning and execution. This includes spending time to seek out experienced and trustworthy educators for training both physicians and care-teams at nursing homes on the ways that they can identify and handle elder abuse. Additionally, whether it is providing additional education or offering expansive resources to support specific cases of elder abuse, organizations must be prepared to allot the appropriate funds needed to support these types of initiatives. Nevertheless, these challenges can be overcome and should be prioritized for the sake of mitigating elder abuse.

## Conclusions

Elder abuse is a complex, multifaceted problem that stems from many underlying issues. Despite government intervention, abuse continues to remain a rampant issue in society with studies showing approximately 10% of people over the age of 65 experiencing some form of abuse. Physicians and other healthcare professionals have a unique opportunity to be able to intervene in such cases but are ill-equipped to do so with the current training they receive. As a result, health care professionals should receive more formal training, either in medical school or during residency, in order to increase their confidence in detecting suspected cases of abuse. Additionally, there are more complex issues at hand, such as fear of violating physician-patient trust and widespread misinformation about reporting cases. While these are far more difficult to address, we must take the first steps in the right direction by educating our health care professionals. As awareness about this issue increases, these secondary barriers may begin to be addressed through increased knowledge and further investigation.
